# RGS3L allows for an M_2_ muscarinic receptor-mediated RhoA-dependent inotropy in cardiomyocytes

**DOI:** 10.1007/s00395-022-00915-w

**Published:** 2022-03-01

**Authors:** Magdolna K. Levay, Kurt A. Krobert, Andreas Vogt, Atif Ahmad, Andreas Jungmann, Christiane Neuber, Sebastian Pasch, Arne Hansen, Oliver J. Müller, Susanne Lutz, Thomas Wieland

**Affiliations:** 1 Experimental Pharmacology Mannheim (EPM), European Center for Angioscience (ECAS), Medical Faculty Mannheim, Heidelberg University, Ludolf-Krehl-Str. 13-17, 68167 Mannheim, Germany; 2 Department of Pharmacology, Center for Heart Failure Research, Institute of Clinical Medicine, University of Oslo and Oslo University Hospital, Oslo, Norway; 3 Department of Cardiology, Heidelberg University, Heidelberg, Germany; 4 Department of Experimental Pharmacology and Toxicology, University Medical Center Hamburg-Eppendorf, Hamburg, Germany; 5 Institute of Pharmacology and Toxicology, University Medical Center Göttingen, Göttingen, Germany; 6 Medical Department III, Christian-Albrechts-Universität Zu Kiel (CAU), Kiel, Germany; 7 DZHK, German Center for Cardiovascular Research, Partner Sites Göttingen, Heidelberg/Mannheim and Hamburg/Kiel/Lübeck, https://dzhk.de

**Keywords:** Cardiac contractility, p190RhoGAP, RacGAP, Regulator of G protein signaling, RhoA

## Abstract

The role and outcome of the muscarinic M_2_ acetylcholine receptor (M_2_R) signaling in healthy and diseased cardiomyocytes is still a matter of debate. Here, we report that the long isoform of the regulator of G protein signaling 3 (RGS3L) functions as a switch in the muscarinic signaling, most likely of the M_2_R, in primary cardiomyocytes. High levels of RGS3L, as found in heart failure, redirect the G_i_-mediated Rac1 activation into a G_i_-mediated RhoA/ROCK activation. Functionally, this switch resulted in a reduced production of reactive oxygen species (− 50%) in cardiomyocytes and an inotropic response (+ 18%) in transduced engineered heart tissues. Importantly, we could show that an adeno-associated virus 9-mediated overexpression of RGS3L in rats in vivo, increased the contractility of ventricular strips by maximally about twofold. Mechanistically, we demonstrate that this switch is mediated by a complex formation of RGS3L with the GTPase-activating protein p190RhoGAP, which balances the activity of RhoA and Rac1 by altering its substrate preference in cardiomyocytes. Enhancement of this complex formation could open new possibilities in the regulation of the contractility of the diseased heart.

**Supplementary Information:**

The online version contains supplementary material available at 10.1007/s00395-022-00915-w.

## Introduction

G-protein-coupled receptors (GPCR) and their regulators are involved in numerous signaling processes in cardiovascular physiology including heart rate, conduction velocity, contractility, and vascular tone [82]. Perturbations in GPCR signaling contribute to pathological developments, such as heart failure, which is one of the leading causes of morbidity and mortality worldwide [67]. Despite evidence for the existence of all five muscarinic acetylcholine receptor subtypes in the heart, the M_2_ muscarinic acetylcholine receptor (M_2_R) is the quantitatively dominant isoform in cardiac tissues [9, 22, 88].

M_2_Rs are known as inhibitory receptors of cardiac function by reducing heart rate and conduction velocity. In addition, they counteract the β-adrenoceptor-mediated increase in contractile force (accentuated antagonism) [25, 42]. Several studies have investigated the effect of vagal nerve stimulation in animal heart failure models or in human heart disease [29, 34, 44]. While reducing the heart rate is protective to the damaged heart and thus aimed for in heart failure therapy, a long lasting negative inotropic response due to M_2_R activation in the ventricles [55] would be detrimental. Moreover, at least in the healthy rats, a chronic infusion of the muscarinic acetylcholine receptor agonist carbachol sensitizes the myocardium to cAMP-induced arrhythmia, most likely by reducing the amount of pertussis toxin (PTX)-sensitive G_i_ proteins in cardiomyocytes [18, 63]. Interestingly, however, activation of the M_2_R caused a Rho-associated protein kinase (ROCK)-mediated inotropic response in an experimental model in the rat based on coronary artery ligation induced infarct and heart failure as well as in the neonatal rat heart [30, 40].

M_2_R signaling is under the control of various regulatory proteins. Regulators of G Protein Signaling (RGS) belong to a diverse family of GTPase-activating proteins (GAP), capable of accelerating GTP hydrolysis of the Gα subunit of heterotrimeric G proteins. Consequently, they are important negative regulators of canonical GPCR signaling pathways. RGS proteins have emerged as potential therapeutic targets due to their function in cardiovascular physiology and pathology [89, 94]. RGS3, which belongs to the R4 RGS protein subfamily, exists in several splice variants. RGS3L, a long isoform of RGS3 (519 amino acids), has in addition to the RGS domain an extended N-terminal segment, which enables interactions with other proteins, such as Gβγ-dimers. Therefore, RGS3L acts not only as a GAP for the Gα proteins, but is also able to regulate the Gβγ-mediated signaling by acting as a Gβγ scavenger [73]. Several RGS3 isoforms, including RGS3L, are expressed in the human heart [56] and RGS3L mRNA and protein levels were upregulated in human heart failure [61]. Fibroblast growth factor 2 (FGF2), a known cardioprotective stimulus, increases RGS3L expression in rat cardiomyocytes [96]. Our group has reported previously, that RGS3L, besides its GAP activity towards G_i_ protein, is able to switch the M_2_R signaling from Rac1 to RhoA activation in the model of M_2_R expressing human embryonic kidney (HEK) cells as well as neonatal rat cardiomyocyte derived H10 cells [87]. However, the underlying molecular mechanism causing this switch is still unclear and the role of RGS3L in native cardiomyocytes has not been studied.

Like heterotrimeric G-proteins, monomeric GTPases of the Rho family, including Rac1 and RhoA, are active in the GTP-bound conformation and inactive in the GDP-bound state. Besides guanine nucleotide exchange factors (GEFs), GAPs are similarly important regulators of monomeric GTPases. GAPs largely accelerate the intrinsic GTPase activity of the small G proteins, and as a result terminate their signaling [86]. p190RhoGAP, a 190 kDa GTPase-activating protein expressed in the heart [72], is a regulator that balances Rac1 and RhoA activities in the cell [26, 71, 72, 90]. Although first described as a GAP specific for RhoA, it became evident that p190RhoGAP can also exhibit GAP activity towards Rac1 and even switch its substrate preference between RhoA and Rac1 [39, 41, 46].

We there performed an explorative study, which will provide evidence that the RGS3L expression level determines whether carbachol stimulation preferentially activates Rac1 or RhoA in cardiomyocytes by inducing an RGS3L/p190RhoGAP complex formation, which switches the GAP activity towards Rac1 and thus increases the amount of active RhoA. We will further demonstrate that this mechanism allows for the observed ROCK-mediated inotropic response upon carbachol stimulation in the rat ventricle.

## Materials and methods

### Antibodies, reagents, and inhibitors

We used the following primary antibodies: mouse-anti-Rac1 (BD Transd. Laboratories, 610650), mouse-anti-p190A (BD Transd. Laboratories, 610149), mouse-anti-RhoA (26C4, Santa Cruz, sc-418), mouse–anti-RGS3 (CC-Q7, Santa Cruz, sc-100762), rabbit–anti-Tiam1 (C-16, Santa Cruz, sc-872), rabbit–anti-Gβ (T-20, Santa Cruz, sc-378), rabbit–anti-RGS3 (Abcam, ab2564), mouse–anti-c-myc (Klon 9E10, Oncogene), mouse–anti-β-actin (Sigma-Aldrich, A2228), mouse–anti-RhoA-GTP (Biomol). The corresponding horseradish peroxidase-conjugated secondary antibodies were from Sigma-Aldrich (Saint Louis, MO, USA, A-9044, A-9169), the fluorescent labelled secondary antibodies (anti-mouse-Alexa Fluor® 568-antibody, anti-rabbit-Alexa Fluor^®^ 633-antibody) were from Life Technologies. In this study, the following reagents and inhibitors were used: FGF-2 (Promega), carbamoylcholine chloride (carbachol), 5-bromo-2′-deoxyuridine (5-BrdU) (Sigma-Aldrich), PI3K-inhibitor LY294002 (2-(4-morpholinyl)-8-phenyl-1(4H)-benzopyran-4-one hydrochloride; Alexis), pertussis toxin (PTX; Calbiochem), H1152P ((S)-(+)-2-Methyl-1-[(4-methyl-5-isoquinolinyl)sulfonyl]homopiperazine, 2HCl, Merck).

### Animal experiments and cell isolation from animals

All the experiments were performed according to the EU animal experiments guidelines and ethical approval by the local German ethics committee (G237/12, Regierungspräsidium Karlsruhe).

### Isolation and culture of neonatal rat cardiomyocytes (NRCM)

NRCM were isolated from hearts of 1–3-day-old male and female neonatal Wistar rats as described previously [92]. Briefly, hearts were minced and subjected to serial digestion in a mixture of collagenase (0.5 mg/ml collagenase type II, Cell systems) and pancreatin (0.6 mg/ml, Sigma-Aldrich) to release single cells. The obtained cell suspension was placed on top of a Percoll gradient (GE Healthcare) to separate cardiomyocytes from other cell types. The cardiomyocyte fraction was seeded on collagen I-coated plates and cultured in DMEM supplemented with 10% (w/v) fetal calf serum, 2 mM l-glutamine, 100 units/ml penicillin and 100 μg/ml streptomycin in a humidified atmosphere of 5% CO_2_ at 37 °C. 0.1 mM 5-BrdU was used to prevent overgrowth of other, non-cardiomyocyte cell types. The cells were used for experiments between 3 and 5 days after isolation. Serum-reduced conditions (DMEM supplemented with 0.5% FCS) were used for 48 h when indicated.

### Cell culture and transfection

HEK-293 cells were grown in Dulbecco’s modified Eagle's medium (DMEM, Invitrogen) supplemented with 10% (w/v) fetal bovine serum, 2 mM l-glutamine, 100 units/ml penicillin and 100 μg/ml streptomycin in a humidified atmosphere of 5% CO_2_ at 37 °C. Transient overnight transfection of the cells with p190RhoGAP, full length RGS3L-N460A, RGS3L or truncated RGS3L constructs were performed using Polyfect (Qiagen) transfection reagent according to the manufacturer's instructions. Cells were incubated under serum-reduced conditions and investigated 24–48 h after transfection. The measurement the RacGAP and RhoGAP activity of p190RhoGAP was performed using a pulldown assay described below.

The HEK-293 cells were from Agilent and routinely monitored for possible mycoplasma contamination.

### Generation of recombinant adenoviruses

The coding sequences of RGS3L and RGS3L-N460A were subcloned into the adenoviral shuttle vector pAdTrack-CMV (a gift from Dr. B. Vogelstein, Baltimore, MD). The cDNA oligonucleotide 5′-ATCCCGGAAGGAATCCTTTTCAGGTTCAAGAGAACTGAAAAGGAT-TCCTTCCTTTTTGGAAA-3′ encoding the RGS3L-shRNA sequence was subcloned into the BglII/HindIII sites of the pShuttle H1 vector (Stratagene). For knockdown of Tiam1 the cDNA oligonucleotide 5′GATCCCGCGAGCTTTAAGAAGAAACTTCAAGAGAGTTTCTTCTTAA-AGCTCGCTTTT-GGAAA-3′ encoding Tiam1-shRNA was used. The complete H1 promoter driven expression cassette was subcloned into pAdTrack [12]. After recombination of the created shuttle vectors with pAdEasy-1, the Pac I linearized recombinant adenoviral genome was transfected and amplified in HEK-293 cells.

### Generation of AAV-9 viruses

The cDNA sequences of the variant/mutant RGS3L-N460A was cloned into a single-stranded AAV genome plasmid (pSSV9-CMV-MLC1500-luc) via XbaI restriction replacing the luciferase (luc) gene. The correct fragment size and orientation was controlled by agarose gel-electrophoresis and sequencing resulting in pSSV9-CMV-MLC1500-RGS3L-N460A. AAV9 vectors were generated by double-transfection of helper plasmid pDP9rs [79] and either pSSV9-CMV-MLC1500-RGS3L-N460A or -luciferase (as control). Vectors were then purified using iodixanol step gradient ultracentrifugation followed by genomic titer determination using quantitative real-time PCR as described previously [31].

### 2D gel electrophoresis

Cell lysates (about 50 µg protein) were mixed with 70 µl rehydration buffer and applied to 7 cm IPG gel strips (GE Healthcare) containing a linear 3–10 pH gradient. Isoelectric focusing was carried out using an Ettan IPGphor unit (GE Healthcare). The subsequent SDS–PAGE was performed on 8% polyacrylamide gels.

### Immunoblot analysis

For immunoblot analysis the protein samples were separated by SDS–PAGE using 8–15% denaturating acrylamide gels and transferred onto nitrocellulose membranes. Membranes were blocked with Roti-Block (Carl Roth) for 1 h at room temperature, and incubated with specific primary antibodies (see above) overnight at 4 °C and according to the manufacturer’s recommendations. After incubation with appropriate secondary antibodies for 1 h, proteins were visualized by enhanced chemoluminescence using an imaging system (Alpha Innotech). Image J Software was used for the analysis of the blots.

### Rac and RhoGTPase activation assay

The cellular Rac1-GTP and RhoA-GTP levels were measured with a pull-down assay using GST fusion proteins containing the Rho-binding domain of rhotekin (GST–RBD) or Rac-binding domain of p21-activated kinase (PAK1) (GST–PBD). GST–RBD and GST–PBD were expressed and purified from *E. coli.* NRCM were stimulated with FGF2 or transduced with adenoviruses and pretreated with the different inhibitors described above. After activation of the NRCM with carbachol (1 mM, 5 min), cells were lysed in ice-cold GST-Fish buffer [11, 87] and pelleted by centrifugation (12,000 rpm, 3 min at 4 °C). The GTP-bound RhoGTPases contained in the supernatant was incubated for 1 h at 4 °C with either GST–PBD bound to magnetic glutathione–sepharose beads or GST–RBD bound to nonmagnetic glutathione–sepharose beads. The beads were separated using a magnetic plate and centrifugation. After twice washing of the beads, bound proteins were eluted with sample buffer and separated by SDS–PAGE. The amounts of activated GTPases and total GTPases from lysates as loading control were then determined by immunoblot analysis as described above.

### Measurement of reactive oxygen species (ROS) in NRCM

NRCM were cultured on 96-well plates (Sarstedt). The cells were transduced with shEGFP, shTiam or RGS3L–N460A encoding adenoviruses, and treated with FGF2 or PTX as described above. Thereafter, the cells were stimulated with 1 mM carbachol or solvent for 5 min at 37 °C. Myocytes were then loaded for 30 min with 5 µM 5(6)-carboxy-2′,7′-dichlorofluorescein (DCF-DA, Sigma-Aldrich) at 37 °C. After washing the cells with HEPES buffered salt solution (HBSS; 25 mM HEPES pH 7,4, 120 mM NaCl, 5,4 mM KCl, 1,8 mM CaCl_2_, 25 mM NaHCO_3_, 15 mM Glucose), DCF fluorescence was measured using the Envision 2102 Multilabel Reader with a set of FITC filters (excitation 485 ± 10 nM and emission at 535 ± 20 nM, PerkinElmer).

### MLC-2 phosphorylation

NRCM were transduced with EGFP (control) or RGS3L–N460A encoding adenovirus for 24 h, treated with 100 nM H1152P for 1 h as indicated and stimulated with 1 mM carbachol or solvent for 90 s at 37 °C. Thereafter, the cells were lysed in a buffer containing phosphatase inhibitor PhosStop (Roche Applied Science). The lysates were cleared by centrifugation and subjected to SDS–PAGE and immunodetection. Finally, densitometric band intensities of pMLC-2 were quantitated using total MLC-2a as control.

### Immunoprecipitation

NRCM were transduced with RGS3L–N460A adenovirus for 24 h and stimulated with 1 mM carbachol or solvent for 5 min at 37 °C. Co-immunoprecipitation was performed as described previously [5]. Briefly cells were lysed with immunoprecipitation buffer (50 mM Tris–HCl, pH 7.4, 2 mM EDTA, 150 mM NaCl, 0.1% SDS, 1% Nonidet P-40, 10 mM NaF) containing 1 mM sodium orthovanadate, 1 mM Pefablock, 10 µg/ml aprotinin, 10 µg/ml leupeptin). After centrifugation the cleared lysates were incubated with the indicated antibodies (2 μg) under agitation for 1 h at 4 °C. After addition of 40 μl 1:1 (v/v) protein-A–sepharose-beads (Amersham Biosciences), the mixture was gently shaken for an additional 3–4 h at 4 °C. Beads were washed three times with immunoprecipitation buffer and eluted in SDS-containing buffer for 5 min at 95 °C. After SDS–PAGE and transfer to nitrocellulose membranes, immunoprecipitated proteins were detected by Western blot analysis using the indicated antibodies according to standard protocols. Final detection was done with an ECL system (Amersham), band intensity was quantified with ImageJ-software.

### Proximity ligation assay (PLA)

Proximity ligation assay was performed by following the manufacturer’s protocol (Olink Bioscience, Uppsala, Sweden). Briefly, NRCM seeded on collagen-coated coverslips in 12-well dishes were stimulated with or without carbachol for 5 min, washed 3 times with PBS, fixed in 4% formaldehyde in PBS for 10 min, permeabilized in 0.02% Triton-X-100 for 10 min, and blocked with Blocking Solution (Olink) for 30 min in 37 °C. After incubation with the indicated primary antibodies (mouse–anti-p190RhoGAP, rabbit–anti-RGS3) over night at 4 °C, wells were washed with Wash Buffer (Olink), incubated with PLA Probe anti-mouse plus and PLA Probe anti-rabbit minus for 1 h at 37 °C. After washing, a ligation and amplification step followed using the manufacturer’s protocol and reagents. Cells were mounted with Duolink In Situ Mounting Medium with DAPI, dried in room temperature and visualized by confocal microscopy (Leica, Germany). The LAS-X software was used for image processing. Quantification of fluorescent signals was performed using Image J software.

### Measurement of the GAP activity of p190RhoGAP towards Rac1 and RhoA

Measurement of the functional RhoGAP activity of p190RhoGAP was performed by a pulldown assay as described previously [5, 58]. This assay is based on the principle that the functional activation of p190RhoGAP is indicated by an increased ability to associate with its substrate, the active, GTP-bound form of monomeric GTPases. The ability of p190RhoGAP to act as a RhoAGAP was detected using constitutively active RhoAQ63L-GST coated beads. The more p190RhoGAP protein binds to the beads, the higher is its RhoAGAP activity (corresponding to a decreased level of RhoA-GTP in the cell [58]). Using RacQ61L-GST-coated beads instead the Rac1GAP-activity of p190RhoGAP can be measured. Briefly, cells (NRCM or HEK-293 cells) were washed carefully with PBS and lysed in a buffer containing 50 mM Tris–HCl, pH 7.4, 10 mM MgCl_2_, 150 mM NaCl, 1 mM DTT, 1% Triton X-100, 10 g/ml each of aprotinin and leupeptin, 1 mM Pefabloc and 1 × Phosstop (Roche). After centrifugation for 3 min at 12,000×g, the supernatants were incubated for 60 min at 4 °C with glutathione–sepharose magnetic beads coated with RhoQ63L or RacQ61L conjugated with GST and purified previously from Rosetta *E. coli* bacteria. The beads were separated using a magnetic plate. After three times washing of the beads, bound proteins were eluted with sample buffer and separated by SDS–PAGE. p190RhoGAP was detected by immunoblotting.

### Immunocytochemistry

Subconfluent NRMC were cultured on 12 well plates (Sarstedt), washed three times with PBS and fixed with 3% paraformaldehyde/PBS for 15 min at room temperature. After the cells were treated with 0.05% Triton-X-100 for 3 min at room temperature and 0.5% bovine serum albumine (BSA) for 45 min at 4 °C, they were incubated with the appropriate antibodies for 16 h at 4 °C. After washing with PBS, the cells were incubated with the indicated secondary antibodies for 1 h at room temperature. Images of the cells mounted at room temperature in PBS were acquired using fluorescence microscopy (Olympus IX 81).

## Isolation and culture of adult mouse ventricular cardiomyocytes (AMVCM)

Ventricular myocytes of adult mice were isolated by retrograde Langendorff perfusion using an enzyme composition of collagenase type I and II, dispase (Liberase DH, Roche) and trypsin. The protocol for the isolation of adult ventricular cardiomyocytes was modified from Borner et al. [8]. The mice (age of 10–20 weeks) were anesthetized with 2% isoflurane in oxygen and sacrificed by cervical dislocation. After fixation on a Styrofoam plate and disinfection with 70% ethanol, the thorax was opened. The heart was isolated by cutting distal from the heart, close to the aortic arch and was directly transferred into ice-cold perfusion buffer. The aorta was cannulated with a buffer-filled modified 20G cannula. The heart was connected to the pre-heated (37 °C) perfusion system with a flow of 3.5 ml per min and washed for 30 s. After perfusion with 29.6 mL of digestion buffer, the ventricles were separated from the atria, and cut into 1–2 mm^3^ pieces in 2.5 ml digestion buffer. Digestion was stopped with 2.5 ml stopping buffer 1 and the tissue was titrated with a wide opening syringe. Undigested tissue was removed by sedimentation, and the isolated cells were resuspended in 4.75 ml stopping buffer 2. Recalcification was performed by in five steps until a final concentration of 960 µM CaCl_2_ was reached. The cells were allowed to sediment by gravity and were then resuspended in fresh pre-warmed adult mouse cardiomyocyte medium. The cardiomyocytes were plated in droplets on laminin-coated glass cover slips (Ø 18 mm) in a 12-well plate or seeded in uncoated 6-well plates and were incubated for 30 min at 37 °C and 5% CO_2_. Unattached cells were removed, and fresh medium was added. The attached cardiomyocytes were cultured for 24 to 48 h at 37 °C and 5% CO_2_. (Buffers and media: Stock perfusion buffer 10 × pH 7.4, 1.13 M NaCl, 47 mM KCl, 6 mM KH_2_PO_4_, 6 mM Na_2_HPO_4_ × 2 H_2_O, 12 mM MgSO_4_ × 7 H_2_O, 100 mM KHCO_3_, 100 mM HEPES; Perfusion buffer 1 x:10% (v/v) Stock perfusion buffer 10 x, 23 mM NaHCO_3_, 30 mM taurine, 5.5 mM glucose, 9.9 mM BDM; liberase solution: 0.4% (w/v) liberase DH in ddH_2_O; Digestion buffer: 29.6 ml Perfusion buffer 1 x, 3.75 µl CaCl_2_ 100 mM, 450 µl liberase solution, 200 µl Trypsin 2.5%; Stopping buffer stock solution: 1% (w/v) BSA 10 mL, Perfusion buffer 1 x; Stopping buffer 1: 2.25 ml Stopping buffer, 1.25 µl CaCl_2_ 100 mM; Stopping buffer 2: 4.75 ml Stopping buffer. 1.9 µl CaCl 100 mM; Culture medium AVMCM MEM, 0.25% (v/v) (−)-blebbistatin (0.5 mM), 1% (w/v) BSA, 1% (v/v) P/S, 1% (v/v) l-glutamine (200 mM), 1% (v/v) ITS-X, laminin solution: 0.01% (w/v) in AMCM-medium).

### Measurement of the RhoA activity in adult mouse cardiomyocytes

Measurement of the RhoA-activity in AVMCM was performed by immunofluorescence staining using anti-RhoA–GTP-antibody, microscope image acquisition, and quantification of fluorescence. Cells isolated from mouse hearts were seeded on collagen-coated coverslips in 12-well dishes in Modified Eagle Medium (MEM) supplemented with 1% BSA, 100 units/ml penicillin and 100 μg/ml streptomycin, 1% l-glutamine, 1% (−) blebbistatin, 1% ITS (insulin–transferrin–selenium) in a humidified atmosphere of 5% CO2 at 37 °C. Cells were transduced with Myc-tagged RGS3L–N460A–adenovirus or control EGFP–adenovirus for 48 h. After stimulation with or without 1 mM carbachol for 5 min, cells were fixed in 4% formaldehyde in PBS for 10 min, permeabilized and blocked for 10 min in a blocking buffer containing 10% FCS, 0.2% Triton-X 100 in Ca^2+^- and Mg^2+^-free PBS. Incubation with primary antibodies (mouse–anti-RhoA–GTP-antibody 1:500, rabbit–anti-c-myc-antibody 1:100) was performed in blocking buffer over night at 4 °C. After washing with PBS, cells were incubated with the corresponding fluorochrome-labeled secondary antibodies (anti-mouse-Alexa Fluor 568-antibody 1:1000, anti-rabbit-Alexa Fluor 633-antibody 1:1000) over night at 4 °C, and washed thereafter with PBS. DAPI (1:1000 in PBS for 1 h) was used to detect nuclei. After washing with PBS and mounting, coverslips were dried at room temperature. Cells were visualized by confocal microscopy (Leica, Germany), software LAS-X was used for image processing. Analysis of the fluorescence intensity of the Alexa Fluor 568 conjugate was performed using Image J (Version 1.47v).

### Preparation of EHT and measurement of the contractility of EHT

Neonatal rat engineered heart tissues (EHT) were generated and analyzed as previously reported [24]. In brief, agarose casting molds were prepared in 24 well plates (NUNC) with liquid agarose (2% (w/v), PBS) and polytetrafluorethylen (PTFE) spacer. After agarose solidification PTFE spacer were removed and polydimethylsiloxan (PDMS) were placed on the 24 well plate so that pairs of flexible PDMS posts reach into each agarose casting mold. Dissociated neonatal rat heart cells were re-suspended in a fibrinogen solution (5 mg/ml). 97 µl were mixed with 3 µl thrombin aliquot (100 U/ml) and pipetted into a casting mold. This pipetting step was repeated for each EHT. Fibrin polymerization took place in an incubator (2 h) and PDMS racks with fibrin gels attached to the PDMS posts were transferred to new 24 well plates. EHT were cultivated in maintenance media (DMEM, Horse serum 10% (v/v), insulin 10 μg/ml, aprotinin 33 μg/ml) with media replacement on Mondays, Wednesdays and Fridays. After development (days 25–30) EHT were transferred to modified Tyrode’s solution containing 0.1 mM free Ca^2+^ (120 mM NaCl, 22.6 mM NaHCO_3_, 5.4 mM KCl, 5 mM glucose, 1 mM MgCl_2_, 0.4 mM NaH_2_PO4, 0.1 mM CaCl_2_, 0.05 mM Na_2_EDTA, 25 mM HEPES) and force analysis was performed by video-optical recording of EHT shortening as recently described [24].

### Injection of the AAV9 virus and preparation of rat ventricular muscle strips

The care and experimental use of all animals in this study were in accordance with institutional guidelines and approved by the local ethics committee (Regierungspraesidium Karlsruhe).

We injected 6-week-old male Wistar rats with AAV9–CMV–MLC1500–RGS3L–N460A-virus or with an AAV9–CMV–MLC1500-luc control virus (10^12^ vg/rat) intravenously. After 2 months, the rats were anaesthetized (2–3% isoflurane flow) and subsequently euthanized by cervical dislocation. The hearts were harvested and mounted on a Langendorff rig perfused with a relaxing solution containing 118.3 mM NaCl, 3 mM KCl, 0.2 mM CaCl_2_, 4 mM MgSO_4_, 2.4 mM KH_2_PO_4_, 24.9 mM NaHCO_3_, 10 mM glucose and 2.2 mM mannitol [77]. The left ventricle was exposed and posterior left ventricular papillary muscles and strips of left ventricles (approx. 1 mm diameter) were excised and mounted in organ baths (Radnoti organ bath System, ADInstruments) containing the relaxing solution (32 °C) as described above and oxygenated (95% O_2_, 5% CO_2_).

### Measurement of the contractility of the isolated rat papillary muscle strips

After mounting, the strips were stretched to ~ 3 mN diastolic tension, allowed to relax ~ 5 min and then the relaxing solution was replaced with a solution of identical composition with the exception of 1.8 mM CaCl_2_ and 1.2 mM MgSO_4_ concentrations. The muscle strips were field stimulated at a frequency of 1 Hz with impulses of 5 ms duration and current about 10–20 mA. The isometrically contracting muscles were stretched to the maximum of their length–tension curve. Contraction–relaxation cycles were recorded and analyzed as previously described [75, 76]. Basal contractility was expressed as maximal developed force (*F*_max_, mN). The descriptive parameters at the end of the equilibration period were used as basal (control) values. Inotropic responses were expressed as changes in the maximal development of force ((d*F*/d*t*)_max_). The measurements were based on averaging 10–20 contraction–relaxation cycles. Antagonists of α1-adrenoceptors (prazosin 1 μM) and β-adrenoceptors (timolol 1 μM) were added 90 min before the muscarinic agonist carbachol which was added to the organ bath as a bolus (100 μM). The contractility measurements were conducted at increasing extracellular Ca^2+^-concentrations (ranging from 1.8 to 3.0 mM).

## Statistical analysis

Exploratory data analysis was performed using the GraphPad Prism software (GraphPad Software, Version 6, La Jolla). Data were expressed as mean ± SD. If not otherwise, indicated Student’s *t* test or one-way-ANOVA with Tukey’s multiple comparison test were performed. A *p* value < 0.05 was considered statistically significant.

## Results

### The RGS3L expression level determines RhoGTPase activation in NRCM upon stimulation with carbachol

**Fig. 1:**
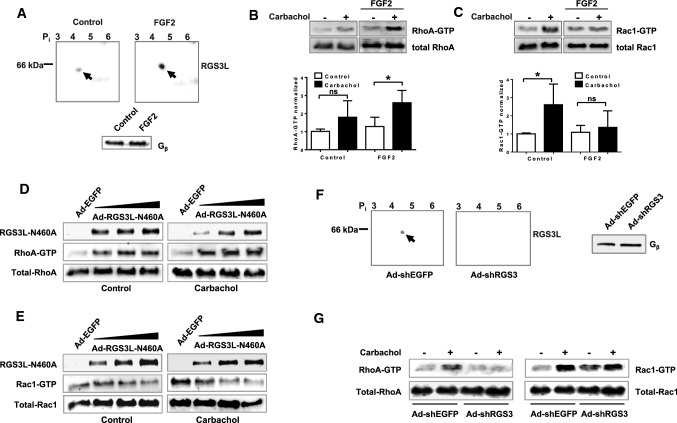
Effect of the expression level of RGS3L on the carbachol-induced RhoGTPase activation in NRCM. **A** Identification of the specific RGS3 isoform expressed in NRCMs treated under serum-free conditions with or without 50 ng/ml FGF2 for 24 h, as indicated. RGS3 in the cell lysates was analyzed by 2D gel electrophoresis and immunoblot. G_β_ was used as a loading control (**B**, **C**). Measurement of the carbachol-induced RhoA (**B**) and Rac1 activation (**C**) in NRCMs without or with FGF2 treatment stimulated without and with 1 mM carbachol for 5 min. Levels of Rac1–GTP and RhoA–GTP were measured by Rac1 and RhoA activation assays. Quantification of Rac1 (Rac–GTP/total Rac1) and RhoA activity (RhoA–GTP/total RhoA) as well as a representative experiment are shown. The mean of Rac1–GTP or RhoA–GTP levels detected in unstimulated cells was set to 1.0. Values are mean + SD (*n* = 8/5 RhoA/Rac1), *p < 0.05. **D**, **E** NRMCs were transduced with Ad-EGFP (Control, MOI 40) and increasing amounts (MOI 10, 20, 30) of Ad-RGS3L–N460A for 48 h. Levels of RhoA–GTP (**D**) and Rac1–GTP (**E**) were determined after 5 min stimulation without or with carbachol. Recombinant RGS3L expression was monitored by immunoblot analysis using the anti-c-myc antibody. **F**, **G** NRCMs were transduced with Ad-shEGFP [12] and Ad-shRGS3L for 72 h. RGS3L expression was monitored by 2D gel electrophoresis and immunoblot. Gβ was used as a loading control. **F** Cells were incubated for 5 min with or without carbachol. Levels of RhoA–GTP and Rac1–GTP were determined

FGF2 increases RGS3 expression in neonatal rat cardiomyocytes (NRCM) [96] and the level of RGS3L determines the M_2_R coupling to Rac1 or RhoA in H10 cells [87]. Therefore, we analyzed RGS3 expression with and without FGF2 treatment and its effect on RhoGTPase activation in NRCM. As established before [87], we used 2D gel electrophoresis with subsequent immunoblotting to identify specific isoforms of RGS3. RGS3L was detected at the predicted isoelectric point (pI) and molecular mass of 4.79 and 61 kDa, respectively (Fig. 1A). In accordance with the published data [91], a marked increase in the amount of RGS3L protein was detected in NRCM treated with FGF2 for 24 h. Next, we examined the activation of Rac1 and RhoA in FGF2-treated NRCM in response to carbachol. Under control condition carbachol caused a prominent Rac1 and a weak RhoA activation, whereas in FGF2-treated cells the opposite was found (Fig. 1B, C). Both the carbachol-induced Rac1 and RhoA activations were reduced by treatment with PTX or the phosphoinositide-3-kinase (PI3K) inhibitor LY294002 independent of FGF2 (Supplemental Fig S1A). These data are in accordance with the G_i_- and PI3K-dependent RhoA and Rac1 activation observed in carbachol-treated NRCM-derived H10 cells and thus indicate the existence of a similar signaling pathway in primary NRCM [87].

To verify the dependence between the expression level of RGS3L and the coordinated activation of either RhoA or Rac1 in NRCM, we altered RGS3L expression using different experimental approaches. First, we transduced NRCM with an adenovirus encoding the GAP-deficient RGS3L mutant RGS3L–N460A, which mediated in M_2_R expressing cells a prolonged carbachol-induced RhoA activation compared to wild-type RGS3L [87]. Increasing expression of RGS3L–N460A in NRCM induced a pronounced RhoA activation in the absence and presence of carbachol (Fig. 1D). In addition, the increasing expression of RGS3L–N460A preferentially suppressed the carbachol-stimulated Rac1 activation (Fig. 1E). Second, loss of function experiments were performed by transducing NRCM with an adenovirus encoding a shRNA directed specifically against RGS3L (Ad-shRGS3L) [15]. As shown in Fig. 1F, the expression of RGS3L was nearly abolished in the Ad-shRGS3L-transduced NRCM compared to cells transduced with a control adenovirus encoding for a shRNA against EGFP. Gβ, which served as a loading control, remained unaffected. Most importantly, RGS3L depletion reduced the carbachol-induced RhoA activation to control level (Fig. 1G). This effect was dependent on the virus load used for NRCM transduction (Supplemental Fig S1B). In contrast, the carbachol-induced Rac1 activity remained unchanged. In summary, these data demonstrate that the expression level of RGS3L dynamically regulates RhoA activation in NRCM and determines whether Rac1 or RhoA activation prevails after carbachol application.

### RGS3L increases carbachol-evoked MLC-2 phosphorylation in a ROCK-dependent manner

**Fig. 2:**
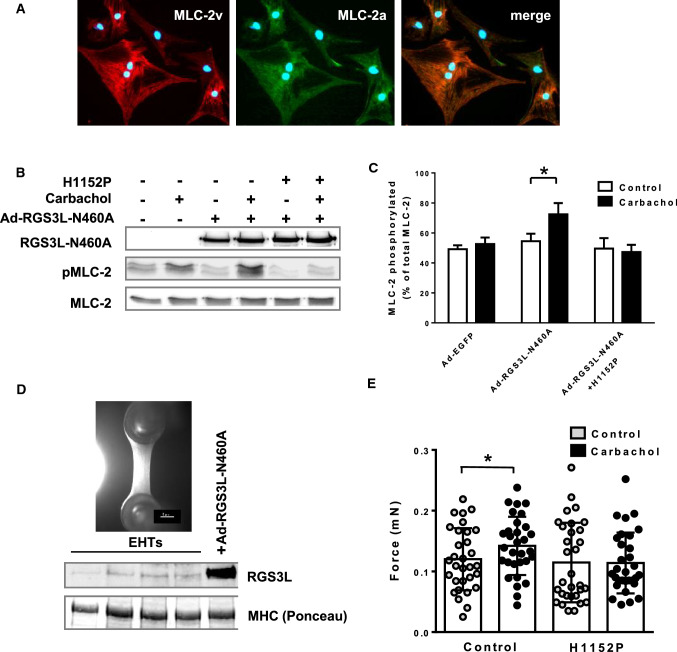
Effect of RGS3L–N460A expression on the carbachol-induced MLC-2 phosphorylation in NRCM and measurement of contractility in rat engineered heart tissue (EHT). **A** Detection of the ventricular isoform MLC-2v and the atrial isoform MLC-2a in NRCMs by immunocytochemistry. **B** NRCMs were transduced with Ad-RGS3L–N460A or Ad-EGFP and stimulated with or without carbachol for 5 min. The phosphorylation status of MLC-2 was analyzed by immunoblot using a polyclonal antibody against MLC-2a-P, which recognizes both, mono- and diphosphorylated (Ser22 and/or Ser23) MLC-2a. MLC-2a was used as a loading control. For ROCK-inhibition, cells were pretreated with 100 nM H1152P for 1 h. **C** Quantification of the MLC-2 phosphorylation in NRCM relative to the total MLC-2 amount. Values are mean + SD (*n* = 4–6), *p < 0.05. **D** Expression of RGS3L in NRCM-derived EHT. Adenoviral overexpressed RGS3L–N460A in NRCM was used as a positive control. **E** EHT were stimulated with 50 μM carbachol with and without treatment with 100 nM H1152P which was added 40 min before stimulation with carbachol. The analysis of the contraction force was performed by video-optical recording of EHT shortening. Statistical analysis was performed by repeated measures ANOVA with Sidak's multiple comparisons test. Values are mean + SD (*n* = 31), *p < 0.05

In the neonatal rat heart as well as in an experimental heart failure model, the M_2_R-induced inotropic response was sensitive to ROCK inhibition and accompanied by increased MLC-2 phosphorylation [30, 40]. In NRCM, both the ventricular isoform MLC-2v and the atrial isoform MLC-2a are present and both localize along the myofilaments (Fig. 2A). To study the influence of RGS3L–N460A on the carbachol-induced MLC-2 phosphorylation, we used a polyclonal antibody against MLC-2a-P, which recognizes mono- and diphosphorylated (Ser22 and/orSer23) MLC-2a. Treatment with carbachol in control-transduced NRCM slightly increased MLC-2 phosphorylation, which was, however, enhanced by the expression of RGS3L–N460A (Fig. 2B). The quantification of the carbachol-induced MLC-2 phosphorylation revealed a 33% increase in the presence of RGS3L–N460A compared to the non-stimulated cells (Fig. 2C). Treatment of the cells with the selective ROCK inhibitor H1152P completely blocked the carbachol-induced MLC-2 phosphorylation in the presence of RGS3L–N460A (Fig. 2B, C).

To study whether the increase in ROCK-mediated MLC phosphorylation is associated with an increase in contractility, rat engineered heart tissues (EHT) were prepared from neonatal rat heart cells. A shown in Fig. 2D, the EHT express detectable levels of RGS3L. In contractility measurements a moderate but statistically significant increase in contractile force was observed after carbachol stimulation (Fig. 2E). This inotropic response was blunted by adding the ROCK inhibitor H1152P (Fig. 2E), which is in accordance with the described carbachol-induced inotropy of the neonatal rat heart [40].

### RGS3L increases the carbachol-induced RhoA activation at the sarcolemma of adult ventricular cardiomyocytes and the contractility in isolated ventricular muscle strips

**Fig. 3:**
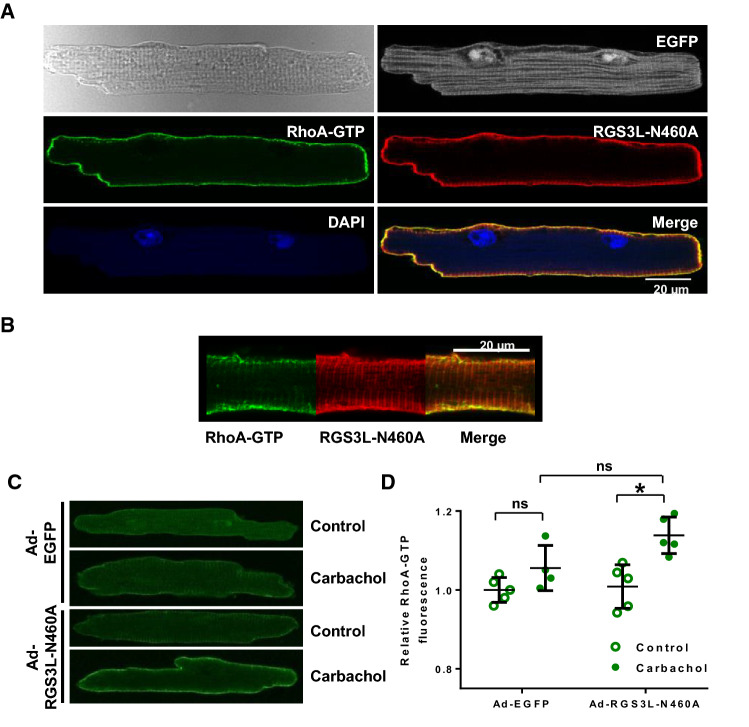
Effect of RGS3L–N460A expression on the M_2_R-mediated RhoA activation in AMVCM. Cells were transduced with Ad-RGS3L–N460A or Ad-EGFP for 48 h. Cells were stimulated with or without carbachol for 5 min. RhoA–GTP was detected with mouse–anti-RhoA–GTP-antibody and secondary anti-mouse-Alexa Fluor^®^ 568-antibody, RGS3L–N460A was detected using rabbit–anti-c-myc antibody and a secondary anti-rabbit-Alexa Fluor^®^ 633-antibody. DAPI was used to detect nuclei. Sarcomeric proteins are visualized by a non-specific incorporation of EGFP. Cells were visualized by confocal microscopy (Leica, Germany) and fluorescence at the sarcolemma was analyzed using ImageJ-software. **A**, **B** Localization of RhoA–GTP and RGS3L–N460A in AMVCM transduced with RGS3L–N460A adenovirus and stimulated with carbachol. Visualization of RhoA–GTP in representative cells (**C**) and analysis of the fluorescence intensity of the Alexa Fluor^®^ 568 conjugate (which corresponds to the RhoA–GTP-amount) at the sarcolemma in 5 transductions obtained from 3 independent AMVCM preparations **(D**). Values are mean + SD, *p < 0.05

NRCMs are a widely used model to study cardiomyocyte signaling, nevertheless, they largely differ from fully differentiated adult cardiomyocytes with regard to myofilament and subcellular organization. Therefore, we studied whether RGS3L–N460A overexpression could also evoke RhoA activation in isolated adult mouse ventricular cardiomyocytes (AMVCM) in which RhoA activation was visualized by confocal microscopy using a specific anti-RhoA–GTP antibody. In addition, the expression of RGS3L–N460A was monitored by immunofluorescence (Fig. 3A, B). Interestingly, we observed that RhoA–GTP and RGS3L–N460A co-localized at the sarcolemma of AMVCM. Moreover, carbachol treatment did not alter the RhoA–GTP level in control transduced AMVCM, but significantly increased it in RGS3L–N460A expressing AMVCM (Fig. 3C, D). These data demonstrate that higher RGS3L expression levels allow for a M_2_R-induced RhoA activation also in adult cardiomyocytes.

**Fig. 4:**
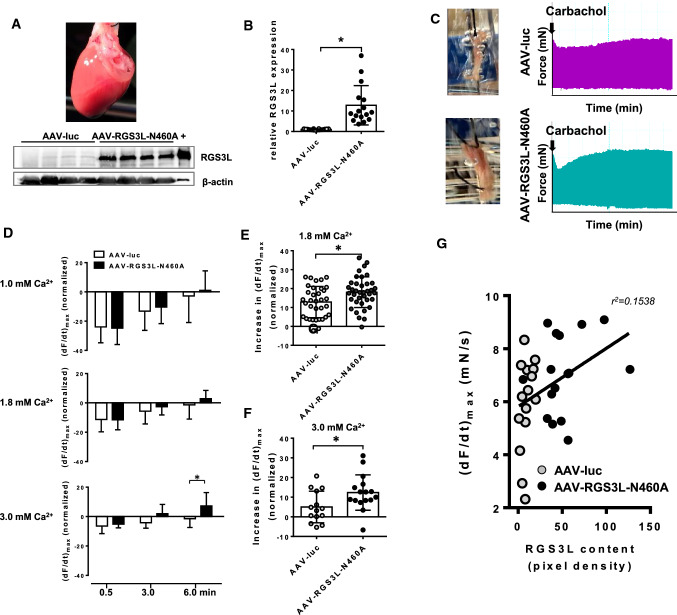
Effect of RGS3L–N460A expression on contractility of rat ventricular muscle strips. **A**, **B** RGS3L-Expression in rat ventricular muscle strips. Male Wistar rats were injected either with an AAV9–CMV–MLC1500-virus encoding RGS3L–N460A or AAV9–ssCMV–MLC1500-luc control virus. The expression of RGS3L in the muscle strips was analyzed after 2 months. A representative blot (**A**) and quantification (**B**) of the relative amount of RGS3L protein compared to the control are shown of *n* = 16/16 muscle strips from 4/4 animals, values are mean + SD, *p < 0.0001, +, lysate of NRCM overexpressing RGS3-N460A. **C** Representative recordings of the inotropic effects of carbachol (100 µM) observed in control transduced and muscle strips overexpressing RGS3L–N460A measured in a Radnoti organ bath System (ADInstrument). **D** Time course (1.5 min, 3 min, 6 min) showing the development of the carbachol-induced inotropic response at different Ca^2+^-concentrations (1 mM, 1.8 mM, 3 mM). Data are shown as the percentage change of (dF/dt)_max_ relative to basal. Values are mean of n = 14–29 muscle strips from 4 to 8 animals + SD, *p < 0.01. **E** Relative increase in the maximal development of force ((d*F*/d*t*)_max_) analyzed between the 0.5 and 6 min timepoints measured in a buffer containing 1.8 mM Ca^2+^. Values are mean of *n* = 34/38 muscle strips form 7/8 animals + SD, *p < 0.01. **F** Relative increase in the maximal development of force ((d*F*/d*t*)_max_) analyzed between the 0.5 and 6 min timepoints measured in a buffer containing 3 mM Ca^2+^. Values are mean of *n* = 34/37 muscle strips + SD, *p < 0.05. **G** Correlation of the maximal contractility (d*F*/d*t*)_max_ 6 min after carbachol-stimulation) and the RGS3L expression of the muscle strips normalized as percentage of a positive control. Values are means of *n* = 16/15 muscle strips from 4/4 animals

We, therefore, aimed to verify that high expression levels of RGS3L in cardiomyocytes allow for a carbachol-induced inotropic response in the healthy ventricular tissue. We injected 6-week-old male Wistar rats either with AAV9–CMV–MLC1500–RGS3L–N460A virus (AAV–RGS3L–N460A) or AAV9–ssCMV–MLC1500-luc control virus (AAV-luc). The animals were housed for a further 2 months prior to experimentation. They did not show any signs of distress and none died. After sacrifice, the hearts were explanted and cardiac contractility was measured ex vivo in ventricular muscle strips (Fig. 4). In line with the data reported before from failing rat hearts [30], carbachol initially decreased the contractility of control as well as RGS3L–N460A expressing muscle strips in a transient manner (Fig. 4C, D). In the RGS3L–N460A-expressing ventricular muscle strips, the return to baseline was accelerated, followed by an inotropic response significantly exceeding the baseline (Fig. 4D). This inotropy was more prominent at higher Ca^2+^ concentrations and later timepoints (Fig. 4D–F). Plotting of the expression level of RGS3L–N460A vs. the measured force of contraction of the individual muscle strip revealed a statistically significant positive correlation between both parameters (Fig. 4G). These results suggest that the expression of RGS3L is indeed linked to the contractile response.

### The RGS3L mediated switch from carbachol-induced Rac1 to RhoA activation requires a complex formation of RGS3L with p190RhoGAP

**Fig. 5:**
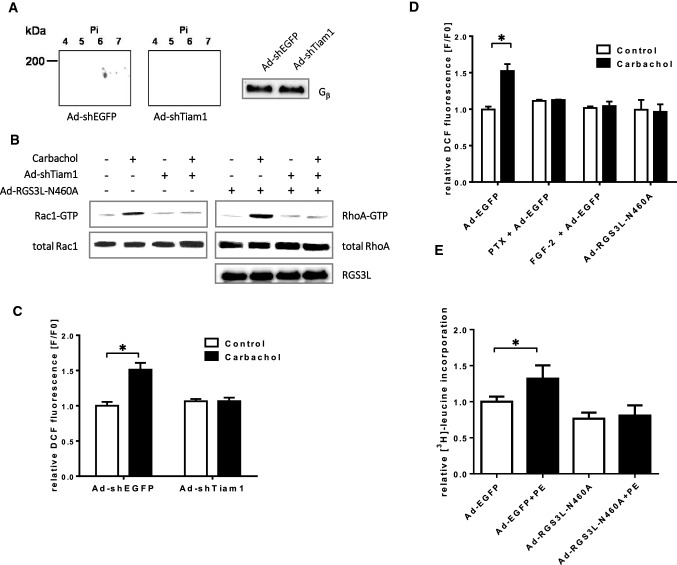
Effect of Tiam1-downregulation and RGS3L–N460A overexpression on carbachol-induced Rac1-activation and Rac1-effector functions NRCMs. Cells were transduced with or without Ad-shEGFP or Ad-shTiam1 for 72 h. **A** Tiam1 expression was monitored by 2D gel electrophoresis and immunoblot. Gβ was used as a loading control. **B** Cells were transduced with Ad-shEGFP, Ad-shTiam1 and Ad-RGS3L–N460A for 72 h as indicated. Levels of RhoA–GTP and Rac1–GTP were determined after 5 min stimulation with or without carbachol. **C**, **D** Quantification of ROS-production in NRCMs by DCF fluorescence. **C** NRCMs were transduced with Ad-shEGFP or Ad-shTiam1 for 72 h. **D** Cells were transduced with Ad-EGFP or Ad-RGS3L–N460A for 24 h and additionally treated with 100 ng/ml PTX or 50 ng/ml FGF2 for 24 h as indicated. Thereafter, the cells were stimulated with carbachol or solvent for 5 min at 37 °C. Values are mean + SD (*n* = 6–10), *p < 0.05. **E** Effect of the RGS3L–N460A-Expression on the Phenylephrine (PE) induced cellular hypertrophy. One μCi/ml [^3^H]–leucine was added into the culture medium shortly after stimulation. After 24 h cellular protein was collected and [^3^H]-content was determined. Measurements were performed in 3 replicates. Values are mean + SD (*n* = 3/6 (EGFP/RGS3L–N460A), *p < 0.05

**Fig. 6:**
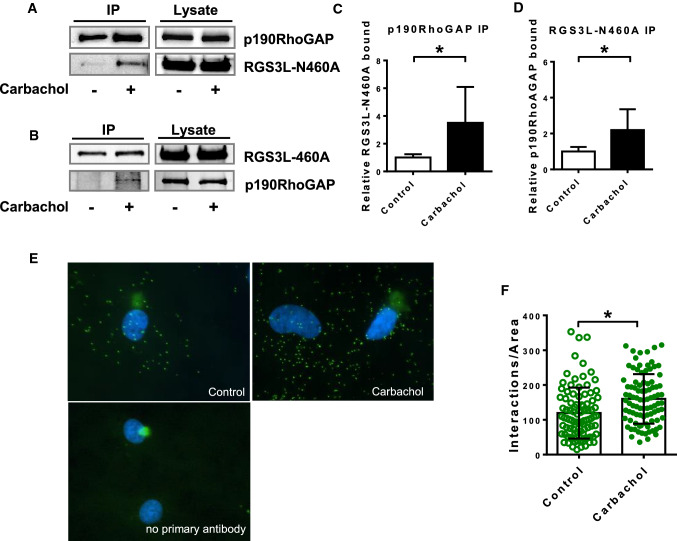
Visualization of the interaction of p190RhoGAP with RGS3L in NRCM. For co-immunoprecipitation experiments cells were transduced with the Ad-EGFP or Ad-RGS3L–N460A and 48 h later, cells were stimulated without and with carbachol for 5 min. Immunoprecipitations were performed using either anti-p190RhoGAP or anti-RGS3 antibodies. Western blots were probed with the corresponding secondary antibodies. Total amount of p190RhoGAP or RGS3L in the cell lysate was used as loading control. Representative experiments (**A**, **B**) and quantification of the relative amount of bound proteins compared to the non-stimulated control (**C**, **D**) are shown of *n* = 11 (**C**) or n = 8 (**D**) experiments. Values are mean + SD, *p < 0.05. **E** Visualization and quantification of the RGS3L–p190RhoGAP interaction using proximity ligation assay, NRCMs were plated on coverslips were stimulated without and with carbachol for 5 min. Phase contrast pictures, DAPI staining and proximity ligation (PLA) were performed. For negative control no primary antibodies were used **F** Quantification of the positive PLA-reactions. The number of the green fluorescent dots per cell was determined for 92 cells from three experiments. Values are mean + SD, *p < 0.05

RGS3L displays no GAP activity towards monomeric GTPases and thus likely influences the activity of RhoGTPases by interacting with either GEFs or GAPs. However, it was shown that its interference with G_i_-mediated Rac1 activation depends on its capacity to bind Gβγ dimers [87]. Previous studies reported that in NRCM the G_i_βγ/PI3K-induced activation of Rac1 via G_i_-protein-coupled receptors is mediated by the guanine nucleotide exchange factor Tiam1 [21, 85]. To investigate the role of Tiam1 in the carbachol-induced Rac1 as well as RhoA activation, we transduced NRCM with an adenovirus encoding a Tiam1-specific shRNA and studied the activation of RhoA and Rac1. As shown in Fig. 5, the expression of Tiam1 was largely reduced compared to cells transduced with a control shRNA encoding virus. The downregulation of Tiam1 abolished the carbachol-induced Rac1 activation (Fig. 5B) as well as the carbachol-induced ROS production (Fig. 5C), which likely originates from the Rac1-dependent activation of NADPH oxidase in NRCM [1]. Consistent with this interpretation, the carbachol-induced production of ROS was sensitive to PTX treatment, RGS3L–N460 overexpression, and FGF2, all suppressing the carbachol-induced Rac1 activation (Fig. 5D). In line with these data, the phenylephrine (PE)-induced protein synthesis, which requires a Giβγ- and PI3K-dependent, Tiam1-mediated Rac1 activation [85], was suppressed by the overexpression of RGS3L–N460A (Fig. 5E). These data suggest that RGS3L could additionally protect the heart not only by reducing the Rac1 activity induced by carbachol, but also by interfering with other Rac1-dependent pathways involved in the development of cardiac hypertrophy. This interpretation is further supported by data showing that the hypertrophy induced by the inflammatory stimulus PGE2 through a Tiam1/Rac1-dependent activation of the transcription factor MEF2 could also be suppressed by RGS3L [83]. Interestingly, the depletion of Tiam1 also abolished the carbachol-induced RhoA activation in the presence of RGS3L–N460A (Fig. 5B), indicating that Tiam1 is also essential in this pathway. Nevertheless, Tiam1 is a bona fide RacGEF. An exchange activity against RhoA has never been reported in a cellular context but it is a well-known protein–protein interaction partner integrating a variety of signaling proteins [7, 50]. As GAPs are as important for Rac1 and RhoA signaling as GEFs, we postulated that p190RhoGAP, which was reported to alter its substrate specificity from RhoA to Rac1 under certain conditions [39, 41, 46], is part of a putative complex and might be responsible for the observed changes in the presence of RGS3L. We, therefore, performed reciprocal co-immunoprecipitation experiments in NRCM transduced with the RGS3L–N460A encoding adenovirus. In line with our hypothesis, p190RhoGAP could be co-immunoprecipitated from NRCM lysates with an anti-RGS3 antibody. Similarly, RGS3L–N460A was co-immunoprecipitated with the anti-p190RhoGAP antibody. Treatment of the NRCM with carbachol before cell lysis increased the amount of the co-precipitated interaction partner by two-to-threefold (Fig. 6A–D). As a weak carbachol-induced RhoA activation also occurred in NRCM in a RGS3L sensitive manner (see Fig. 1E), we studied the p190RhoGAP/RGS3L interaction also in non-transduced NRCM. As these conditions are below the detection level of the co-immunoprecipitation assay, we performed a more sensitive proximity ligation assay (PLA) in NRCM. Thereby, we detected a complex formation already under basal conditions. In accordance with the co-immunoprecipitation assays, carbachol significantly increased the interaction between both proteins, indicating that the carbachol-induced p190RhoGAP/RGS3L complex formation occurs also in native conditions (Fig. 6E, F).

**Fig. 7:**
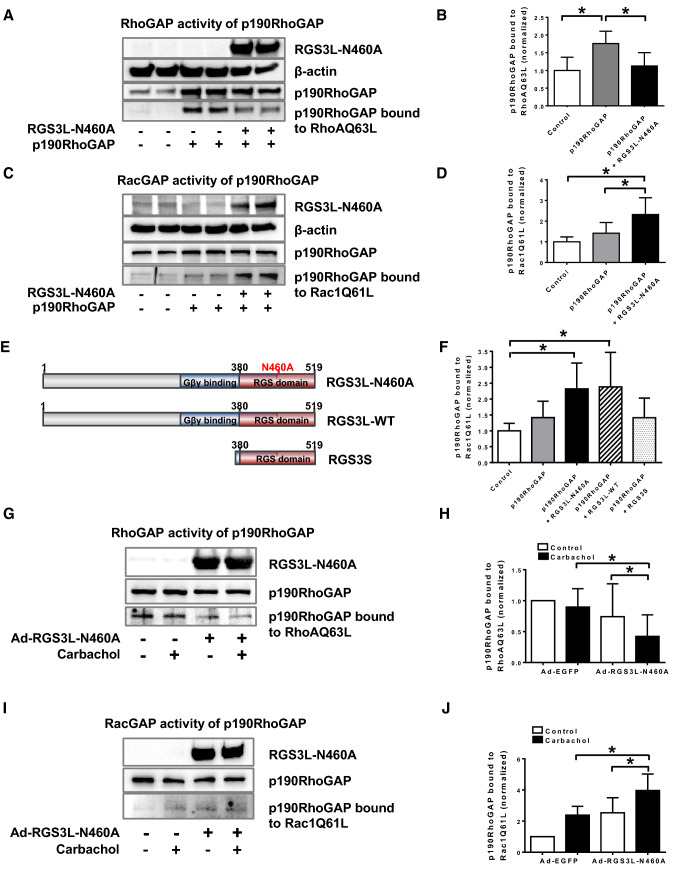
Measurement of the RhoAGAP and Rac1GAP activity of p190RhoGAP in HEK-293 cells and NRCM. **A**–**F** Measurement of the p190RhoGAP-activity in HEK-293 cells expressing p190RhoGAP with or without RGS3L. Functionally active p190RhoGAP was precipitated from the cell lysates with constitutive active RhoAQ63L–GST (**A**, **B**) or constitutive active Rac1Q61L–GST (**C**, **D**) coated beads. Immunostaining with anti-p190RhoGAP-antibody was used to detect p190RhoGAP in the precipitates (active p190RhoGAP) and in total lysates (loading control). Representative immunoblots of experiments performed in duplicate (**A**, **C**) as well as quantification of pixel density of *n* = 5 experiments (**B**, **D**) are shown. p190RhoGAP bound to RhoAQ63L or Rac1Q61L was normalized to the loading control. Values are mean + SD, *p < 0.05. **E** Molecular structure of the used RGS3 isoforms. **F** Measurement of the RacGAP activity of p190RhoGAP in HEK-293 cells expressing the indicated RGS3 isoforms. Values are mean + SD, *n* = 5, *p < 0.05. **G**–**J** Measurement of the RhoAGAP- and Rac1GAP-activity of p190RhoGAP in NRCM. After 48 h transduction with Ad-RGS3L-N460A or Ad-EGFP, cells were incubated with or without carbachol for 5 min. Relative RhoAGAP/Rac1GAP activity of p190RhoGAP was determined by the amount of bound p190RhoGAP to RhoAQ63L- or Rac1Q61L-coated beads normalized to the total amount of p190RhoGAP in cell lysates. Representative experiments (**G**, **I**) as well as quantification of the p190RhoGAP activities (**H**, **J**) are shown. The control value was set to 1.0 in each individual experiment. The other values are shown as mean + SD of *n* = 9 (**H**) or *n* = 7 (**J**) independent experiments. One-way ANOVA with Tukey’s multiple comparison was performed for these values, *p < 0.05

The specificity of a GAP can be monitored by binding to constitutively active monomeric GTPases [58]. Therefore, we studied whether RGS3L expression can alter the substrate specificity of overexpressed p190RhoGAP in HEK cells, the system in which the RGS3L-dependent switch from Rac1 to RhoA was originally characterized [87]. Co-transfection of RGS3L–N460A with p190RhoGAP reduced the binding of p190RhoGAP to the constitutively active RhoAQ63L mutant (Fig. 7A, B) and reciprocally increased the binding to the constitutively active Rac1Q61L mutant (Fig. 7C, D), supporting our hypothesis of a substrate switch of p190RhoGAP. Like the RGS3L–N460A mutant, wild-type RGS3L, but not the N-terminally truncated isoform RGS3S is able to confer RhoA activation [87]. To test whether the switch in GAP activity of p190RhoGAP follows a similar pattern, we co-transfected HEK-293 cells with p190RhoGAP together with RGS3–N460A or RGS3S encoding constructs (Fig. 7E). In accordance with its ability to confer RhoA activation, the change in the GAP activity of p190RhoGAP was only observed in the presence of wild-type RGS3L and its N460A mutant, but not when RGS3S was expressed. This clearly demonstrates that the extended N-terminus of RGS3L is not only required for the Gβγ binding [87], but is also a requisite for the regulation of the GAP activity of p190RhoGAP (Fig. 7F). Next, we determined whether carbachol could change the substrate specificity of endogenously expressed p190RhoGAP for RhoA and Rac1 in the presence of RGS3L in NRCM. Without RGS3L–N460A expression, p190RhoGAP bound predominantly to constitutively active RhoA (Fig. 7G). In contrast, when RGS3L–N460A was expressed, carbachol reduced the binding of p190RhoGAP to RhoAQ63L and increased the binding to Rac1Q61L (Fig. 7G– - J). The switch was detectable after 3 min and more prominent at 5 min (Supplemental Fig S2). Taken together these data clearly indicate that the interaction of RGS3L and p190RhoGAP is essential for re-balancing the activity of Rac1 and RhoA in response to carbachol in cardiomyocytes.

## Discussion

There is increasing evidence that cholinergic signaling could play a protective role in heart failure [36, 38] as the stimulation of the *nervus vagus* could protect the heart from remodeling and improved survival in animal heart failure models. However, whether such an approach is feasible in humans is a matter of debate. The first clinical trials investigating this promising therapeutic approach for human heart failure were not as beneficial as expected [44, 45, 60, 70]. During ischemia/reperfusion after myocardial infarction, however, vagal stimulation improved the outcome in a small cohort study [27, 93]. Nevertheless, muscarinic receptors play an important role in the control of cardiac and vascular functions. The M_2_R is the dominant cholinergic receptor subtype in the heart. However, surprisingly, it is also expressed in ventricular cardiomyocytes lacking parasympathetic innervation [14, 55]. M_2_Rs and G_i_ proteins are upregulated in heart failure [13, 84, 91] and exhibit a protective role in cardiac ventricular function and against the occurrence of cardiac arrhythmias [23, 37, 95]. On the other hand, chronic carbachol infusion sensitized the myocardium to cAMP-induced arrhythmia, most likely by reducing the content of PTX-sensitive G_i_ proteins in cardiomyocytes [18, 63]. Interestingly, it was recently shown that in addition to the parasympathetic regulation, acetylcholine (ACh) is secreted directly from cardiomyocytes [62, 66]. This non-neuronal ACh plays an important protective role in the regulation of myocardial function in both basal as well as in pathologic conditions and might be a possible therapeutic target in cardiovascular diseases [32, 38, 43, 52, 66, 69]. Cardiac ACh protected the heart from remodeling and mice lacking this non-neuronal source of ACh showed increased oxidative stress, remodeling, and hypertrophy [51, 65]. In addition, in heart failure a cholinergic trans-differentiation of the cardiac sympathetic nerves via cytokines secreted from the failing myocardium occurs [19, 33, 59], which might represent another source of ventricular ACh. Therefore, in addition to the correction of the sympathovagal balance in the heart, the so far not fully understood cholinergic signaling through M_2_Rs in ventricular cardiomyocytes might be beneficial in the failing heart, possibly providing a new target for treatment of cardiovascular diseases [32].

**Fig. 8:**
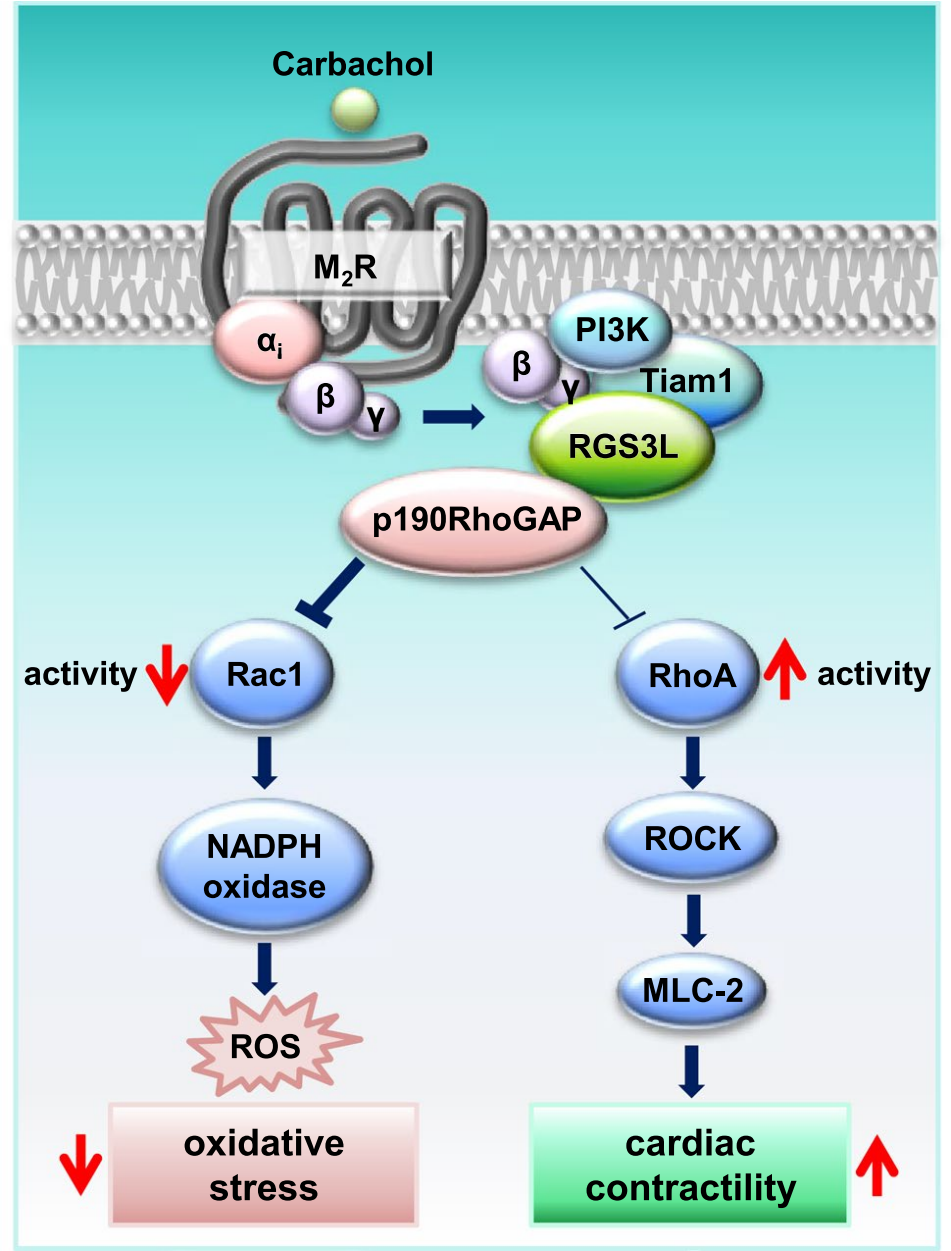
Schematic overview of the RGS3L-mediated switch in M_2_R signaling from Rac1 to RhoA activation and its functional consequences in cardiomyocytes

Based on data obtained from a model of experimental heart failure in rats, which showed a ROCK-mediated inotropic response to M_2_R activation only in failing hearts [30] and a RhoA/ROCK-mediated, carbachol-induced inotropy in neonatal rat hearts upon M_2_R activation [40], we propose that the carbachol-induced RhoA/ROCK-mediated responses are elicited by this receptor subtype. This interpretation is supported by the sensitivity of the carbachol-induced RhoA activation to PTX treatment. In contrast to the G_q/11_-mediated RhoA activation elicited by the M_3_R [47], which might contribute to the regulation of cardiac contractility [35], the M_2_R-induced, G_i/o_-mediated RhoA activation in the presence of RGS3L is PTX sensitive [87]. We, therefore, studied the role of RGS3L in mediating a carbachol-induced RhoA activation in cardiomyocytes (NRCM, AMVM). We obtained evidence that the amount of expressed RGS3L regulates whether a carbachol-induced RhoA activation occurs or not. Whereas the depletion of RGS3L completely abolished the carbachol-induced RhoA activation, increasing its expression level, either by FGF2 treatment or adenoviral expression, strongly enhanced the ability of carbachol to activate RhoA. In accordance with the previously described pathway mediating the M_2_R-induced RhoA activation in HEK cells [87], the carbachol-induced RhoA activation in NRCM was inhibited by PTX and LY294002 treatment, indicating the involvement G_i/o_ proteins and the Gβγ-dependent activation of PI3K (Fig. 8).

Similar to observations in the artificial M_2_R-expressing HEK cell system, the increased carbachol-induced RhoA activation was accompanied by a decrease in carbachol-induced Rac1 activation. G_i/o_-coupled GPCRs, including M_2_Rs, are known to activate Rac1 in a variety of cell types [64]. In cardiomyocytes, the G_i/o_-dependent Rac1 activation is mediated by Gβγ dimers and involves the activation of PI3K and the Rac-GEF Tiam1 [85]. Rac1 activity has essential functions in the heart and is also involved in the development of cardiovascular diseases, e.g., it is involved in the hypertrophic response in cardiomyocytes [10, 68, 85]. Moreover, Rac1 is an essential co-factor of certain NADPH oxidases, which induce through increased oxidative stress cardiac dysfunction and injury [17, 48, 49, 57]. Thus, mice expressing constitutively active Rac1 developed cardiomyopathy and exhibited a post-ischemic contractile dysfunction [80, 81]. Therefore, inhibition of Rac1 signaling by enhanced RGS3L expression is a potential cardioprotective therapeutic strategy in cardiac hypertrophy, fibrosis, arrhythmias, and heart failure [3, 20]. In line with this interpretation, upregulation or overexpression of RGS3L in NRCM suppressed the carbachol-induced ROS production, the phenylephrine-induced hypertrophic protein synthesis in NRCM, and hypertrophy induced by the inflammatory stimulus PGE2 [83]. In addition, the RGS3L upregulation likely interferes with the detrimental G_i_βγ-mediated, muscarinic receptor induced activation of p38 mitogen-activated protein kinases, acting upstream of ROS production [28, 53, 73].

Interestingly, the signaling pathway used by M_2_Rs to increase RhoA activity in the presence of RGS3L (see Fig. 8 and [87]) is similar or even identical to that used to activate Rac1 in the absence of RGS3L. This interpretation is supported by the data obtained by PTX treatment, PI3K inhibition, and Tiam1 depletion, which similarly abolished, both carbachol-induced RhoA and Rac1 activity observed with and without RGS3L–N460A expression, respectively. As mentioned before, Tiam1 does not induce an activation of other monomeric GTPases than Rac1 in living cells [21, 85]. Therefore, it is likely that the switch from Rac1 to RhoA activation in the presence of RGS3L is regulated by another molecule, most likely attracted to the same signaling complex by binding to Gβγ/RGS3L. A potential candidate was p190RhoGAP as it is expressed in cardiomyocytes and most importantly can switch its substrate preference from RhoA to Rac1 [39, 41, 46]. In general, the regulation of the GAP activity can occur by several ways, such as autoregulation, lipid-binding, protein-binding, phosphorylation, or changing the subcellular localization [4, 16, 41, 46, 54, 74]. Moreover, by a recent meta-analysis, which aimed to evaluate sequence–structure–function relationship of different RhoGAPs and Rho proteins, it was suggested that the RhoGAP domain itself is rather nonselective and under cell-free conditions it can be even inefficient [2]. Instead, other domains of RhoGAPs can determinate the substrate specificity and fine-tune the catalytic efficiency of the GAP domain in the cell. In accordance with that notion, we obtained evidence that RGS3L is able to form a complex with p190RhoGAP and thereby its substrate preference switched from RhoA to Rac1. In line with this hypothesis, the downregulation of the endogenous RGS3L expression increased the basal activity of Rac1 (see Fig. 1G). Most importantly, the complex formation in cardiomyocytes is regulated by muscarinic receptors, thus likely accounting for the loss in carbachol-induced Rac1 activity and simultaneously increasing RhoA–GTP levels (Fig. 8). Taken together, our data demonstrate an interesting new mechanism involving an M_2_R-induced switch which regulates the Rac1–RhoA balance in cardiomyocytes, and could account for the M_2_R-induced increased cardiac contractility seen in experimental heart failure and neonatal rat hearts [30, 40]. In line with this hypothesis, we observed an enhanced carbachol-induced phosphorylation of MLC-2 upon expression of RGS3L–N460A which was sensitive to ROCK inhibition.

The ex vivo contractility measurements of ventricular muscle strips from rats in which RGS3L–N460A was expressed by a cardiotrophic AAV construct [6] revealed a positive correlation between the RGS3L–N460A expression level and an increase in contractility and allowed for carbachol-induced inotropy in a Ca^2+^-dependent manner. This Ca^2+^-dependency fits well to the effects known of increased RhoA and ROCK activation in cardiomyocytes, which allows for an increased phosphorylation of MLC through inhibition of MLC-phosphatase at higher Ca^2+^ concentrations within the cell [78].

As this muscarinic inotropic mechanism bona fide bypasses cAMP signaling it might be part of the beneficial effects of non-neuronal ACh in heart failure together with the afore discussed decrease in Rac1-dependent ROS production and cardiomyocyte hypertrophy. Therefore, it will be interesting to determine if there is therapeutic potential of AAV-mediated RGS3L–N460A expression in experimental heart failure models.

## Conclusions, study limitations and further perspectives

In this study we combined several in vitro models, NRCM, AMVCM, and HEK-293 cells, to clarify the mechanism how G_i/o_-coupled muscarinic receptors, which are based on our previous work most likely of the M_2_ subtype ([30, 40, 87], can induce RhoA activation dependent on the expression of RGS3L. Our data demonstrate a G_i/o_-dependent complex formation of RGS3L with p190RhoGAP, which likely depends on the known interaction of RGS3L with Gβγ dimers [73, 87], that switches the GAP activity of p190RhoGAP from RhoA to Rac1. As a consequence, the agonist-induced Rac1 activity is decreased, whereas the RhoA activity is increased.

NRCM and AMVCM are from different species and largely differ regarding their differentiation, compartmentalization, and fetal vs. mature gene expression. HEK-293 cells are even regarded as an artificial cellular model. Thus, the finding that the G_i/o_/RGS3L dependent signaling can be observed in different species and developmental stages indicate a rather common signaling pathway, which generally regulates the activation of RhoGTPases, e.g., by M_2_Rs, dependent on the expression level of RGS3L in cardiomyocytes and is already present in early developmental stages. In line with this interpretation an increase in RhoA activity caused by carbachol stimulation was, as far as studied, always accompanied by a reduction in Rac1 activation.

The M_2_R-induced, RhoA/ROCK-mediated increase in cardiac contractility was first described in left ventricular strips isolated from adult rat hearts developing heart failure after myocardial infarction [30], To test the hypothesis, that the RGS3L expression level is crucial to carbachol induced inotropy, we choose a model in which a cardiomyocyte specific RGS3L overexpression can be achieved in vivo and the effect of this overexpression could be studied under similar experimental conditions as described before [30]. Although, an RGS3L-dependent, carbachol-induced increase in contractility could be demonstrated, this model has limitations in its significance. For example, it cannot predict whether RGS3L overexpression has a realistic chance to be developed into a therapeutic intervention for the treatment of heart failure. To study this, the RGS3L overexpression strategy has to be tested for example in mouse or rat heart failure models and analyzed in vivo, e.g., by echocardiography to assess its influence on cardiac contractility. In addition, the second likely beneficial effect of the RGS3L overexpression, the inhibition of the detrimental activation of Rac1-dependendent signaling, which herein was only preliminary characterized in NRCM, can be analyzed in such models in more detail. We are, therefore, pursuing such a strategy in our laboratory.

## Supplementary Information

Below is the link to the electronic supplementary material.

Supplementary file1 (PDF 294 KB)
