# Supplementary material for: RGS3L allows for an M2 muscarinic receptor-mediated RhoA-dependent inotropy in cardiomyocytes
Source: Basic Res Cardiol. 2022 Mar 1;117(1):8. doi: 10.1007/s00395-022-00915-w (PMC8888479; doi:10.1007/s00395-022-00915-w)
Supplement: Supplementary file 1 — Supplementary file1 (PDF 294 KB) [file 395_2022_915_MOESM1_ESM.pdf]

Figure S1

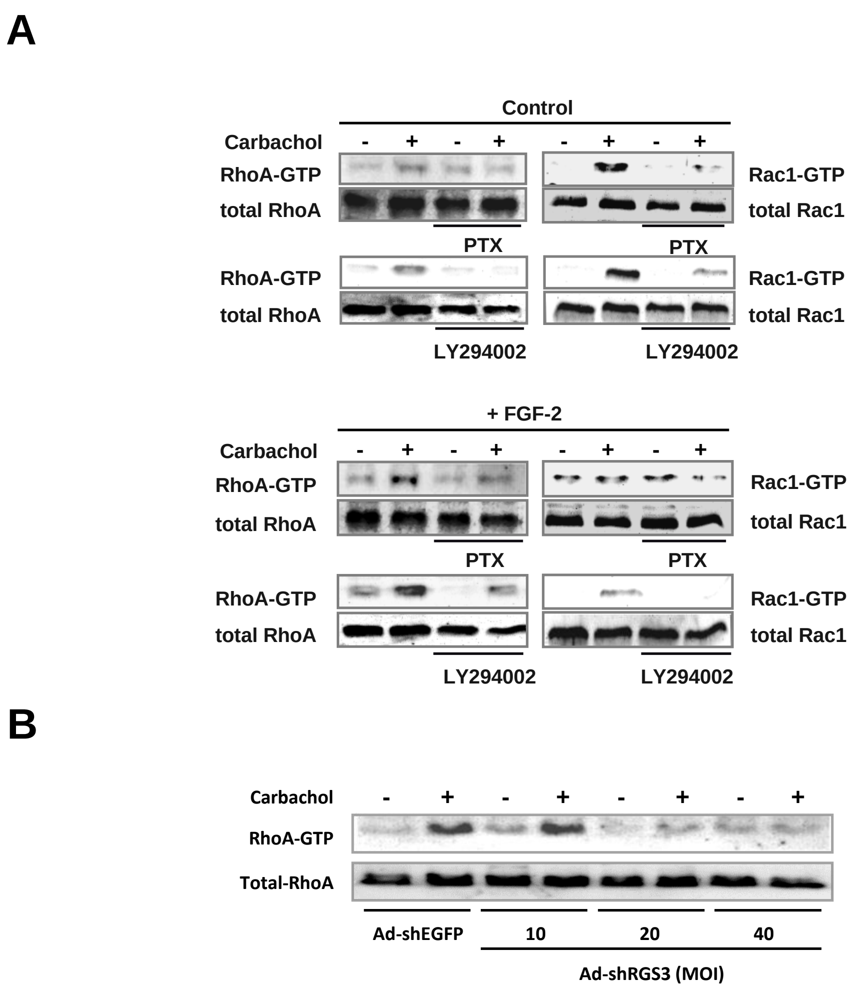

(A) Effect of PTX and LY294002 on the carbachol-induced Rac1 and RhoA activation in NRCM. To increase RGS3L expression, NRCM were incubated with 50 ng/ml FGF2 for 24 h if indicated. For inhibition of G_i-protein and PI3K signaling, cells were treated with 100 ng/ml pertussis toxin (PTX) and 50 µM LY294002 for 2 h before stimulation with carbachol for 5 min, respectively. Levels of Rac1-GTP and RhoA-GTP were measured by Rac1 and RhoA activation assays. (B) NRCMs were transduced either with Ad5-shEGFP (MOI 40) or with increasing amounts of Ad5-shRGS3-shRNA for 72 h. After 5 min stimulation with or without carbachol, RhoA activation was measured. Like in M2R human embryonic kidne

Figure S2

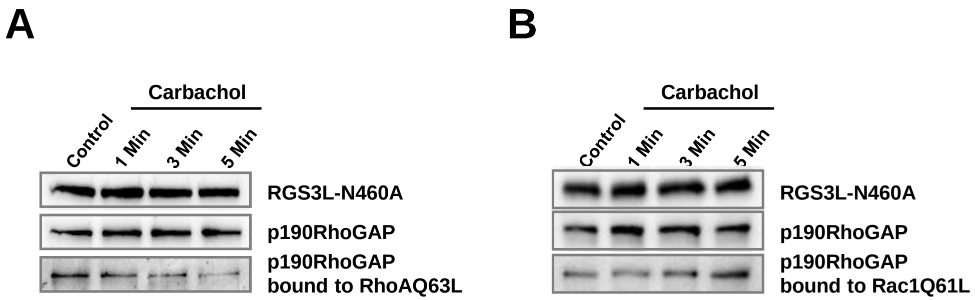

Time dependence of the change in RhoGAP (A) and RacGAP activity (B) of p190RhoGAP after carbachol stimulation in NRCM. Cells were transduced with Ad-RGS3L-N460A for 48h and then stimulated with carbachol for 1, 3 or 5 minutes. Relative RhoGAP and RacGAP activity of p190RhoGAP was determined by the amount of bound p190RhoGAP to RhoAQ63L and Rac1Q61L beads, respectively. The total amounts of p190RhoGAP and RGS3L-N460A in the cell lysates are shown as loading controls.
